# Lactate-induced lactylation in skeletal muscle is associated with insulin resistance in humans

**DOI:** 10.3389/fphys.2022.951390

**Published:** 2022-08-30

**Authors:** Dominic Maschari, Gunjan Saxena, Timothy D. Law, Erin Walsh, Mason C. Campbell, Leslie A Consitt

**Affiliations:** ^1^ College of Health Sciences and Professions, Ohio University, Athens, OH, United States; ^2^ Department of Biomedical Sciences, Ohio University, Athens, OH, United States; ^3^ Ohio Musculoskeletal and Neurological Institute, Ohio University, Athens, OH, United States; ^4^ Biological Sciences Department, Ohio University, Athens, OH, United States; ^5^ Diabetes Institute, Ohio University, Athens, OH, United States

**Keywords:** skeletal muscle, post-translation modification, insulin sensitivity, lactylation, lactate, obesity

## Abstract

Elevated circulating lactate has been associated with obesity and insulin resistance. The aim of the current study was to determine if lactate-induced lysine lactylation (kla), a post-translational modification, was present in human skeletal muscle and related to insulin resistance. Fifteen lean (Body Mass Index: 22.1 ± 0.5 kg/m^2^) and fourteen obese (40.6 ± 1.4 kg/m^2^) adults underwent a muscle biopsy and 2-h oral glucose tolerance test. Skeletal muscle lactylation was increased in obese compared to lean females (19%, *p* < 0.05) and associated with insulin resistance (r = 0.37, *p* < 0.05) in the whole group. Skeletal muscle lactylation levels were significantly associated with markers of anaerobic metabolism (plasma lactate and skeletal muscle lactate dehydrogenase [LDH], *p* < 0.05) and negatively associated with markers of oxidative metabolism (skeletal muscle cytochrome c oxidase subunit 4 and Complex I [pyruvate] OXPHOS capacity, *p* < 0.05). Treatment of primary human skeletal muscle cells (HSkMC) with sodium lactate for 24 h increased protein lactylation and IRS-1 serine 636 phosphorylation in a similar dose-dependent manner (*p* < 0.05). Inhibition of glycolysis (with 2-deoxy-d-glucose) or LDH-A (with sodium oxamate or LDH-A siRNA) for 24 h reduced HSkMC lactylation which paralleled reductions in culture media lactate accumulation. This study identified the existence of a lactate-derived post-translational modification in human skeletal muscle and suggests skeletal muscle lactylation could provide additional insight into the regulation of skeletal muscle metabolism, including insulin resistance.

## Introduction

Elevated blood lactate has been linked to health conditions including cancer (de la Cruz-Lopez et al., 2019) and sepsis (Lee and An 2016), and is considered a risk factor for the development of type 2 diabetes (Juraschek et al., 2013). Fasting lactate levels have been reported to be increased in obese and type 2 diabetics compared to healthy individuals (Reaven et al., 1988; Lovejoy et al., 1992; Crawford et al., 2008), and associated with insulin resistance (Lovejoy et al., 1992; Chondronikola et al., 2018). Furthermore, interventional studies proven to improve insulin sensitivity, including exercise (Chondronikola et al., 2018; Jones et al., 2019), bariatric surgery (Jones et al., 2019), and weight loss (Chondronikola et al., 2018) have reduced blood lactate levels in obese individuals. Taken together, these studies suggest lactate may be a biomarker for metabolic dysregulation, including insulin resistance.

Skeletal muscle, the main source of lactate, plays a vital role in glucose homeostasis with approximately 70–80% of insulin-mediated glucose uptake taking place in this tissue (DeFronzo et al., 1981). Skeletal muscle insulin resistance is considered the primary defect leading to the development of type 2 diabetes (DeFronzo and Tripathy 2009) making skeletal muscle the ideal tissue to identify metabolic defects contributing to insulin resistance. During carbohydrate metabolism, glucose enters the muscle cell and undergoes glycolysis to produce pyruvate. Pyruvate enters the mitochondria to be converted to acetyl-CoA by pyruvate dehydrogenase (PDH) (oxidative metabolism) (45) or is converted to lactate via lactate dehydrogenase (LDH; anaerobic metabolism). Defects in skeletal muscle mitochondria often precede the development of metabolic disease (Petersen et al., 2004; Ritov et al., 2005) and likely contributes to lactate accumulation in obese, insulin resistant individuals when pyruvate production exceeds oxidative capabilities (Avogaro et al., 1996). Similarly, we have reported that older adults with impaired insulin-stimulated skeletal muscle PDH function have elevated plasma lactate when normalized to insulin-stimulated glucose uptake (Consitt et al., 2016). It is speculated that impaired skeletal muscle PDH activity in the elderly results in the preferential shuttling of pyruvate to lactate during hyperinsulinemia. Collectively, these findings suggest that imbalances in pivotal metabolic enzymes contribute to the buildup of lactate, however it remains unclear if the accumulation of this metabolite is simply a consequence of metabolic dysfunction or if it could act as a signaling molecule in skeletal muscle to further elicit metabolic disease.

In 2019, a novel role for lactate was discovered which involves lactate-induced addition of lactyl groups to lysine (K) residues, termed lactylation (Zhang et al., 2019). Early research investigating this post-translational modification focused on the lactate induced-lactylation of histones in cell lines and the subsequent effects on gene transcription. Treatment of MCF-7 cells with conditions to promote lactate via hypoxia or rotenone resulted in increased histone lactylation, whereas treatment of cells with glycolytic inhibitors including 2-deoxy-d-glucose (2-DG) and oxamate, reduced histone lactylation (Zhang et al., 2019). More recently, global lysine lactylome analysis of the fungal pathogen, *Botrytis cinerea*, identified 166 lactylated proteins of which 27% were located within the mitochondria (Gao et al., 2020). Similarly, Meng et al. (Meng et al., 2021) recently reported 342 lactylated proteins in developing rice, with a high concentration of these proteins located within glycolytic and TCA cycle metabolic pathways. Collectively, these studies demonstrate a novel role for lactate as a signaling molecule with possible downstream consequences on gene regulation and metabolism. The purpose of the current study was to utilize both *in vivo* and *in vitro* experiments to determine if lactate-induced lactylation occurred in human skeletal muscle and whether this post-translational modification was associated with insulin resistance in humans.

## Methodology

### Human subjects

Fifteen lean (BMI: 22.1 ± 0.5 kg/m2) and fourteen obese (40.6 ± 1.4 kg/m2) men and women were recruited to undergo an oral glucose tolerance test (OGTT) and skeletal muscle biopsies. Characteristics of the subjects are provided in Table 1. Briefly, all participants were sedentary (participated in less than one hour of organized physical activity per week), nonsmokers, and were not taking medications known to alter carbohydrate or lipid metabolism. Females participated during the follicular phase of their menstrual cycle (days 1–6) and all participants had maintained a constant body mass (±2 kg) in the 6 months before the experimental session. The protocol was in accordance with the Declaration of Helsinki and was approved by Ohio University.

**TABLE 1:** Participant characteristics.

	Lean individuals (n = 15)	Obese individuals (n = 14)
Sex (M/F)	8/7	7/7
Age (years)	19.9 ± 0.7	22.3 ± 1.2
Body Mass Index (BMI)	22.1 ± 0.5	40.6 ± 1.4*
Body Fat (%)	25.7 ± 1.6	43.0 ± 1.0*
Insulin Sensitivity (Matsuda Index)	7.7 ± 1.2	2.4 ± 0.5*

Data are presented mean ± SEM. *p < 0.05 vs lean individuals.

During the study, participants reported to the Clinical Translational Research Unit (CTRU) at Ohio University on two separate occasions. During the first session, subjects provided their informed consent, completed a health questionnaire, and had body composition measured by dual X-ray absorptiometry. On the second visit, participants arrived at the CTRU between 0700 and 0800 after a 12-h overnight fast for the OGTT and muscle biopsies.

### OGTT and muscle biopsies

For the OGTT, a catheter was placed into a peripheral vein and a baseline blood sample was obtained (-5 min). A 75g glucose beverage (Trutol 75, Fisher Scientific) was ingested within 2 min, and blood plasma was obtained every 15 min for a 2-h period and stored at -80 °C for the subsequent analysis of glucose, insulin and lactate. Plasma glucose and lactate were analyzed in duplicate using the YSI 2300 STAT Plus Glucose and Lactate Analyzer (YSI Inc., Yellow Springs, Ohio). Plasma insulin was measured using a human insulin ELISA kit (Millipore, Burlington, MA). The Matsuda Index was calculated from the OGTT and used as a measure of insulin sensitivity. For the current study, a skeletal muscle biopsy was obtained at baseline from the vastus lateralis using the percutaneous needle biopsy technique and immediately trimmed of any visible connective or adipose tissue and placed in either BIOPS buffer (10 mM Ca-EGTA buffer, 0.1 µM free calcium, 20 mM imidazole, 20 mM taurine, 50 mM K-MES, 0.5 mM DTT, 6.56 mM MgCl2, 5.77 mM ATP, 15 mM phosphocreatine, [pH 7.1]) for respiration studies (∼10–15 mg) or frozen in liquid nitrogen for subsequent protein analyses.

### Permeabilized muscle fiber respiration

To further investigate the relationship between lactylation and PDH function, complex I (pyruvate supported) OXPHOS capacity was analyzed in permeabilized skeletal muscle fibers. Due to limited tissue availability, this was only completed in a subset of study participants (lean n = 4, obese n = 4). Mitochondrial respiration was measured using the Oroboros Oxygraph-2K (Oroboros Instruments, Innsbruck, Austria) as previously described (Miotto et al., 2020; Monaco et al., 2021). Briefly, muscle fibers were separated and permeabilized in BIOPS buffer supplemented with saponin (30 µg/mL) and washed in respiration buffer (MiR05; pH 7.0) containing EGTA (0.5 mM), MgCl2·6H2O (3 mM), K-lactobionate (60 mM), KH2PO4 (10 mM), HEPES (20 mM), sucrose (110 mM), and fatty acid-free BSA (1 g/L). Permeabilized fibers were added to MiR05 buffer in the oroboros chambers followed by the addition of 2 mM malate (Sigma-Aldrich, St. Louis, MO), 5 mM pyruvate (Sigma-Aldrich) and 5 mM ADP (Sigma-Aldrich) to determine pyruvate-supported complex I OXPHOS capacity. Respiration rates were normalized by the initial muscle wet weight.

### Primary cultures of human skeletal muscle cells (HSkMCs)

Muscle biopsies (50–100 mg) were obtained from the vastus lateralis of eight women (n = 8) using the percutaneous needle biopsy technique. Satellite cells were isolated, cultured and cryopreserved for subsequent HSkMC experiments, as previously described (Bell et al., 2010; Consitt et al., 2010). For experiments, HSkMCs were thawed on passage 2 or 3 and subcultured onto 6-well type I collagen-coated plates. After reaching approximately 80% confluency, myoblasts were differentiated into myotubes by switching growth media to differentiation media (Dulbecco’s Modified Eagle’s Medium supplemented with 2% horse serum, 0.5 mg/ml BSA, 0.5 mg/ml fetuin, and 50 U/ml penicillin/streptomycin). Data are presented as biological replicates (HSkMC from different individuals).

### Primary human skeletal muscle cell experiments

To determine the effects of lactate on HSkMC insulin resistance, myotubes on Day 5 of differentiation were treated with 20 mM sodium chloride (control) or different concentrations of sodium lactate (0 mM, 10 mM, 20 mM) for 24 h and phosphorylation of IRS-1 on serine residue 636, a marker of insulin resistance (Bouzakri et al., 2003; Vlavcheski and Tsiani 2018; Den et al., 2020; Vlavcheskih et al., 2020), was measured by western blot procedures (described below). Additionally, IRS-1 serine phosphorylation was measured in response to HSkMC LDH-A siRNA treatment (described below).

To determine the effects of lactate on myotube lactylation, a series of experiments were initiated on Day 5 of myotube differentiation. To investigate the direct effects of exogenous lactate on protein lactylation, myotubes were incubated with 20 mM sodium chloride (control) or different concentrations of sodium lactate (0 mM, 1 mM, 10 mM, 20 mM, or 40 mM) for 24 h. To determine the effects of hyperglycemia on HSkMC lactylation and lactate accumulation, myotubes were exposed to media containing low glucose (5.6 mmol/L) or high glucose (25 mmol/L) for 24 h. To determine the effects of glycolysis inhibition on HSkMC protein lactylation and lactate accumulation, myotubes were incubated with 2-DG (0 mM, 5mM, or 10 mM) for 24 h. To investigate the effects of inhibiting the conversion of pyruvate to lactate, myotubes were treated with sodium oxamate (0 mM vs 20 mM), a specific chemical inhibitor of LDH-A.

For all experiments, HSkMC were washed twice with ice-cold PBS on Day 6 and harvested in lysis buffer (50 mmol/L HEPES [pH 7.4], 1% Triton X-100, 10 mmol/L EDTA, 100 mmol/L NaFl, and 12 mmol/L Na pyrophosphate) supplemented with protease and phosphatase inhibitors (Sigma-Aldrich). Cell lysates were stored at -80°C for later protein analysis.

### LDH-A siRNA transfection

On Day 3, primary human myotubes were transfected with 12.5 nM of either validated silencer select siRNA to target LDH-A or Silencer Select negative control (ThermoFisher Scientific, Waltham, MA). Transfections were performed with Lipofectamine RNAiMAX transfection reagent (Invitrogen, Carlsbad, CA) in Opti-MEM reduced serum media (ThermoFisher Scientific), according to manufacturer’s guidelines. After 24 h (Day 4), the medium was removed and replaced with fresh differentiation media. On Day 5 of differentiation, the medium was removed and replaced with fresh differentiation medium (5.6 mM glucose) or differentiation medium with 20 mM glucose for 24 h. On Day 6, cells were harvested as described above.

To provide further evidence that glycolysis and LDH-A were inhibited during the above experiments, culture medium was collected prior to harvesting cells and lactate concentrations determined. Briefly, media was spun at 12,000 x g for 10 min at 4°C, and L-lactate measured with a YSI 2300 STAT Plus Glucose and Lactate Analyzer (YSI Inc., Yellow Springs, Ohio). Lactate concentrations were normalized to myotube total protein per well.

### Western blot (immunoblot) procedures

Skeletal muscle was homogenized in lysis buffer and protein content was determined for both tissue and HSkMC. Western procedures were performed as previously described (Consitt et al., 2008; Consitt et al., 2013; Consitt et al., 2017; Consitt et al., 2018). Briefly, 20 μg of cell lysate were separated by SDS-PAGE, electrotransferred onto polyvinylidene difluoride membranes (Millipore, Billerica, MA) and probed overnight with l-lactyllysine (PTM Biolabs, Chicago, IL), LDH-A (Santa Cruz Biotechnology, Santa Cruz, CA), COXIV (Cell Signaling, Danvers, MA), phosphorylation of IRS-1 (Ser636, Cell Signaling), or Histone H3 (Cell Signaling). Samples were normalized to a control sample on each gel. IRS-1 phosphorylation levels were additionally normalized to IRS-1 total protein (Cell Technology) after membranes were stripped, as previously reported (Consitt, Koves et al., 2016). Non-phosphorylated protein was normalized to tubulin (Cell Signaling). Membranes probed with l-lactyllysine were later stained with Coomassie Blue (Biorad, Hercules, CA).

### Statistics

Analyses were performed using SPSS version 28.0 software (SPSS Inc., Chicago, IL). Pearson correlation coefficients were used to measure the strength of associations between skeletal muscle lactylation and metabolic variables. An unpaired *t*-test was used to compare skeletal muscle lactylation levels between lean and obese individuals. A paired *t*-test was used to determine the effects of sodium oxamate treatment. One-way ANOVA was used to determine the effects of sodium lactate and 2-DG doses on HSkMC lactylation and phosphorylation of IRS-1 on serine 636. A two-way ANOVA was used to determine the effects of LDH siRNA under different glucose doses on HSkMC lactylation and IRS-1 phosphorylation on serine 636. Data are presented as means ± SEM. Statistical significance was defined as *p* < 0.05.

## Results

### Human Skeletal Muscle Lactylation, obesity and insulin sensitivity

**FIGURE 1:**
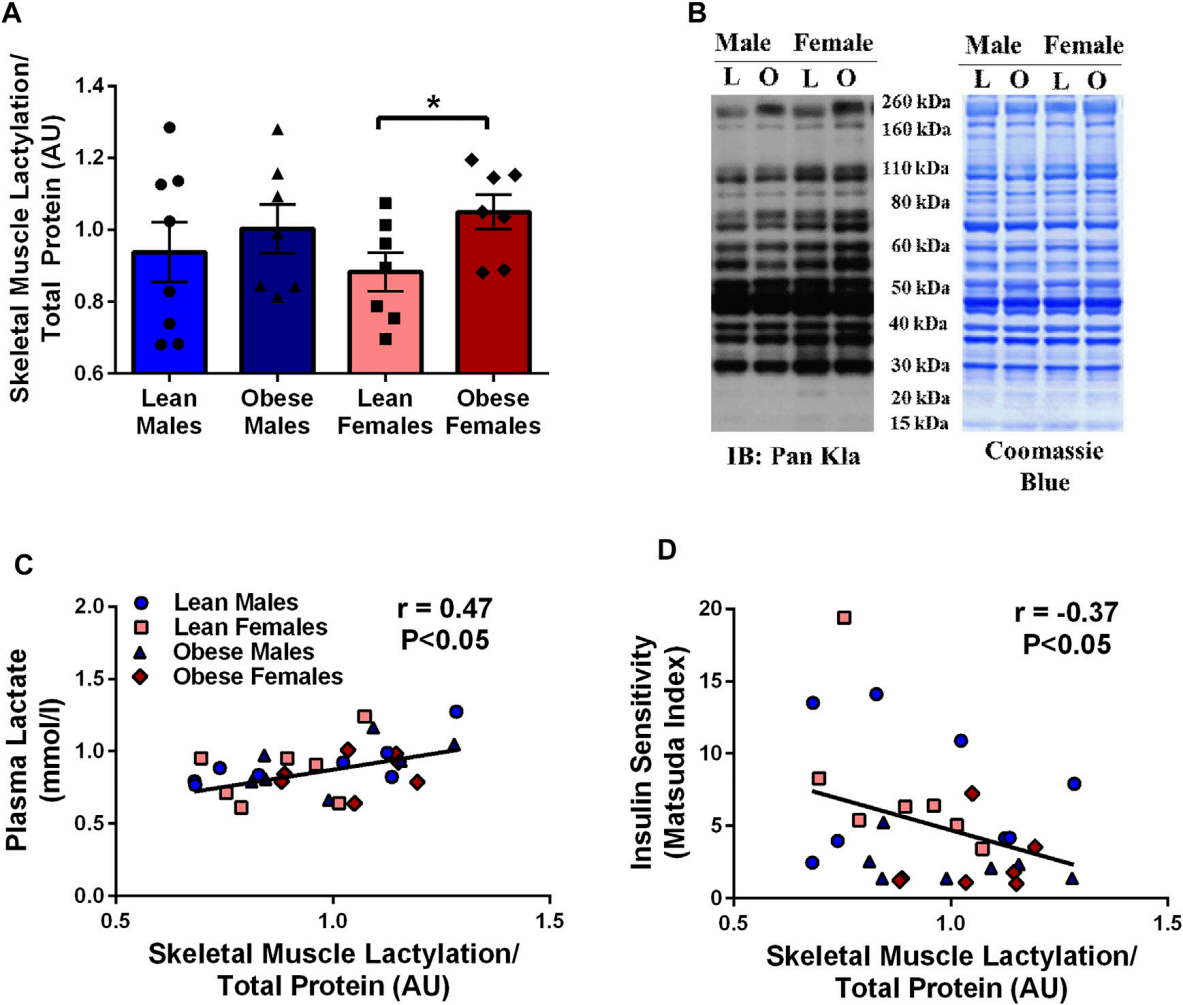
Skeletal Muscle Lactylation in Lean and Obese Individuals. Quantification of skeletal muscle lactylation in lean (male = 8, female = 7) and obese (male = 7, female = 7) individuals **(A)**. Skeletal muscle lactylation levels were normalized to Coomassie Blue (total protein) and presented relative to total protein. *p < 0.05 vs lean females. Human skeletal muscle representative blot for pan-kla and Coomassie blue **(B)**. Relationship of fasting plasma lactate and skeletal muscle lactylation in the whole group (n = 29) **(C)**. Relationship of insulin sensitivity (Matsuda Index) and skeletal muscle lactylation in the whole group (n = 29) **(D)**. In all instances skeletal muscle lactylation levels were normalized to Coomassie Blue (total protein) and presented in arbitrary units (AU). Data is expressed as mean ± SEM. Light blue circles represent data points for lean males; light pink squares represent data for lean females; dark blue triangles represent data points for obese males; dark pink diamonds represent data from obese females.

There was a tendency for obese individuals to have higher (13%, *p* = 0.09) skeletal muscle lactylation levels than lean individuals. When lactylation levels were further analyzed by sex, obese females had higher levels than lean females (19%, *p* < 0.05, Figure 1A). Fasting skeletal muscle lactylation was positively associated with fasting plasma lactate levels in all individuals (r = 0.47, *p* < 0.05, Figure 1C). Insulin sensitivity, as measured by the Matsuda Index was negatively associated with muscle lactylation levels (r = -0.37, *p* < 0.05, Figure 1D) in the whole group.

### Human Skeletal Muscle Lactylation is negatively associated with markers of skeletal muscle oxidative metabolism

**FIGURE 2:**
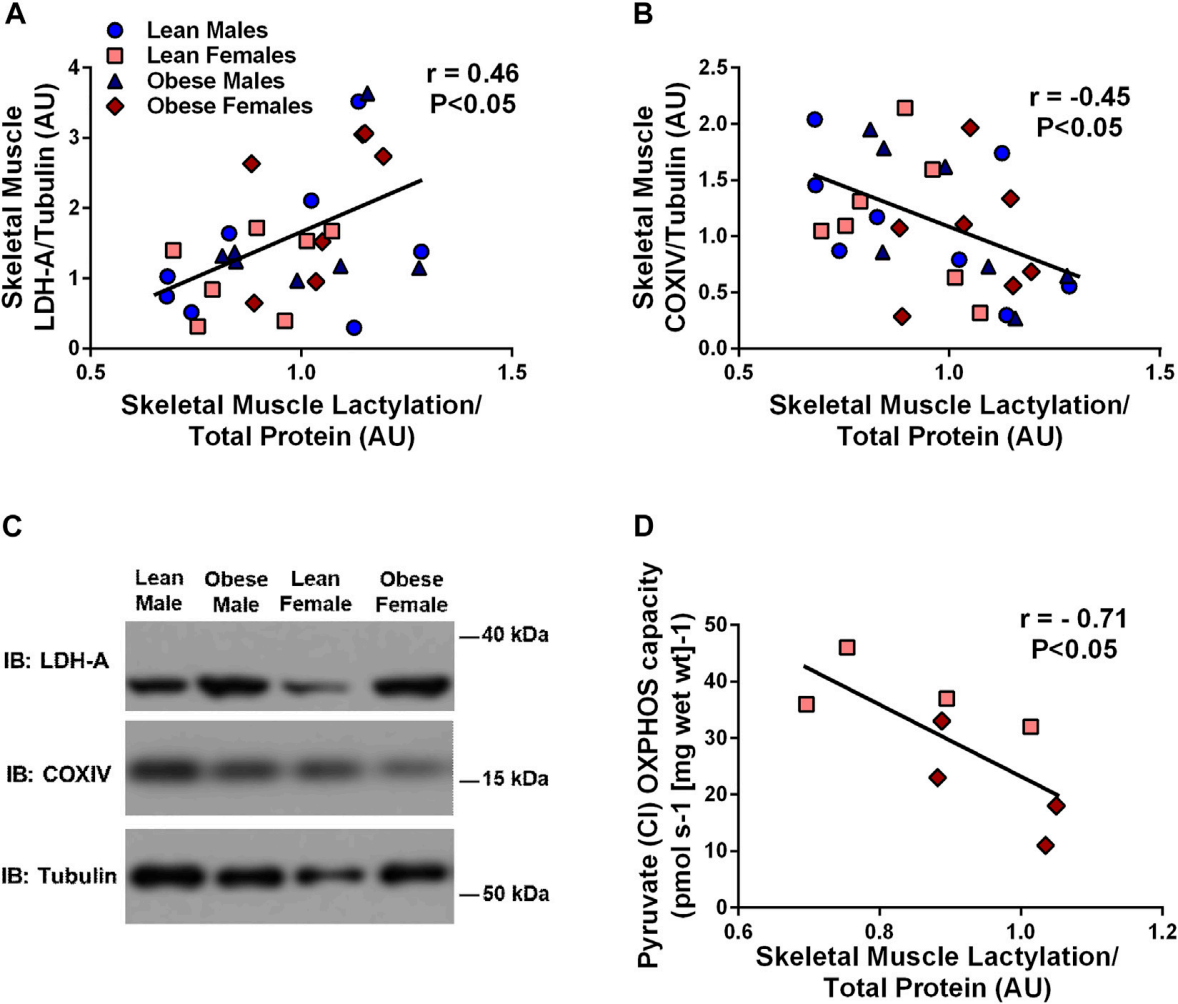
Relationship Between Human Skeletal Muscle Lactylation and Markers of Skeletal Muscle Anaerobic/Oxidative Metabolism. Relationship of skeletal muscle LDH-A protein expression and skeletal muscle lactylation in the whole group (n = 29) **(A)**. Relationship of skeletal muscle COXIV protein expression and skeletal muscle lactylation in the whole group (n = 29) **(B)**. LDH-A and COXIV were normalized to tubulin and lactylation levels were normalized to Coomassie Blue. Representative blots for skeletal muscle COXIV and LDH-A in lean and obese males and females **(C)**. Relationship between skeletal muscle complex I (pyruvate supported) OXPHOS capacity in permeabilized skeletal muscle fibers and skeletal muscle lactylation in subset of study individuals (n = 4 lean, n = 4 obese) **(D)**. All skeletal muscle protein values are presented in arbitrary units (AU). Data is expressed as mean ± SEM. Light blue circles represent data points for lean males; light pink squares represent data for lean females; dark blue triangles represent data points for obese males; dark pink diamonds represent data from obese females.

Skeletal muscle lactylation levels were positively associated with skeletal muscle LDH (r = 0.46, *p* < 0.05, Figure 2A) and negatively associated with the mitochondrial marker, COXIV (r = -0.45, *p* < 0.05, Figure 2B). Complex I (supported by pyruvate) OXPHOS capacity in permeabilized muscle fibers was negatively associated with skeletal muscle lactylation in a subset of study individuals (r = -0.71, *p* < 0.05, n = 8, Figure 2D).

### Lactate-induced IRS-1 serine phosphorylation in primary human skeletal muscle cells

**FIGURE 3:**
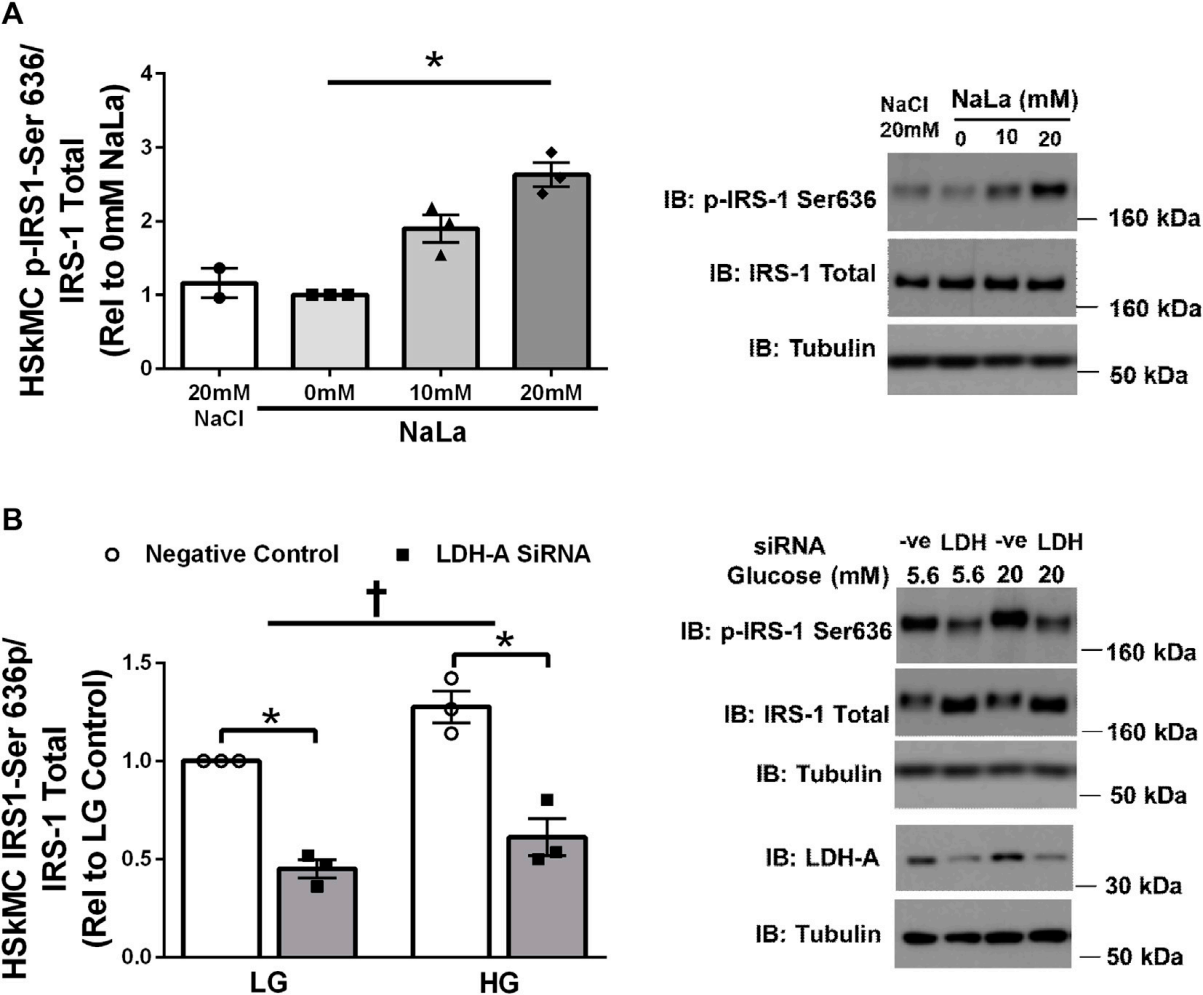
Lactate-Induced IRS-1 Serine Phosphorylation on Site 636 in Primary Skeletal Muscle Cells. Quantification of IRS-1 serine 363 phosphorylation in human primary skeletal muscle cells in response to increasing sodium lactate concentrations (n = 3 for NaLa dose response, n = 2 for NaCl control) with representative blot **(A)**. Quantification of phosphorylation on serine site 636 in HSkMC in response to negative control (Silencer Select) and LDH siRNA under low glucose (LG, 5.6 mmol/L) and high glucose (HG, 20 mmol/L) treatment (*n* = 3) and representative blot **(B)**. Data is expressed as mean ± SEM. *p < 0.05 main effect for inhibitory treatment; †*p* < 0.05 low glucose vs high glucose. Sample size (n) represents biological replicates from HSkMC derived from different individuals.

Treatment of primary HSKMC with lactate for 24 h resulted in an increase in IRS-1 phosphorylation on serine 636 in a dose-dependent manner (*p* < 0.05, Figure 3A). In response to decreased LDH-A expression (∼-55%, *p* < 0.05), HSkMC IRS-1 serine 636 phosphorylation was reduced (∼-48%, *p* < 0.01, Figure 3B), despite elevations in IRS-1 total protein (∼50%, *p* < 0.05, Figure 3B) under both low (5.6 mM) and high glucose (20 mM) culture media concentrations.

### Lactate-induced lactylation in primary human skeletal muscle cells

**FIGURE 4:**
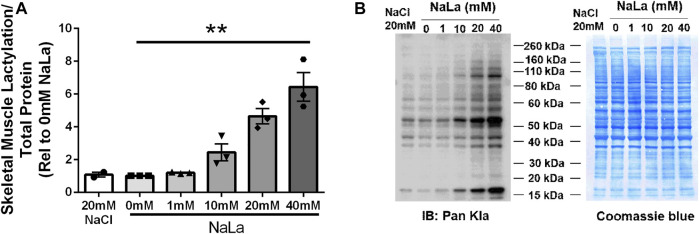
Lactate-Induced Lactylation in Primary Humans Skeletal Muscle Cells. Quantification of lactylation in human primary skeletal muscle cells in response to increasing sodium lactate concentrations (n = 3 for NaLa dose response, n = 2 for NaCl control) **(A)**. Myotube lactylation levels were normalized to Coomassie Blue. Representative blot for pan-kla and Coomassie blue **(B)**. Data is expressed as mean ± SEM. **p < 0.005 main effect for lactate dose. Sample size (n) represents biological replicates from HSkMC derived from different individuals.

**FIGURE 5:**
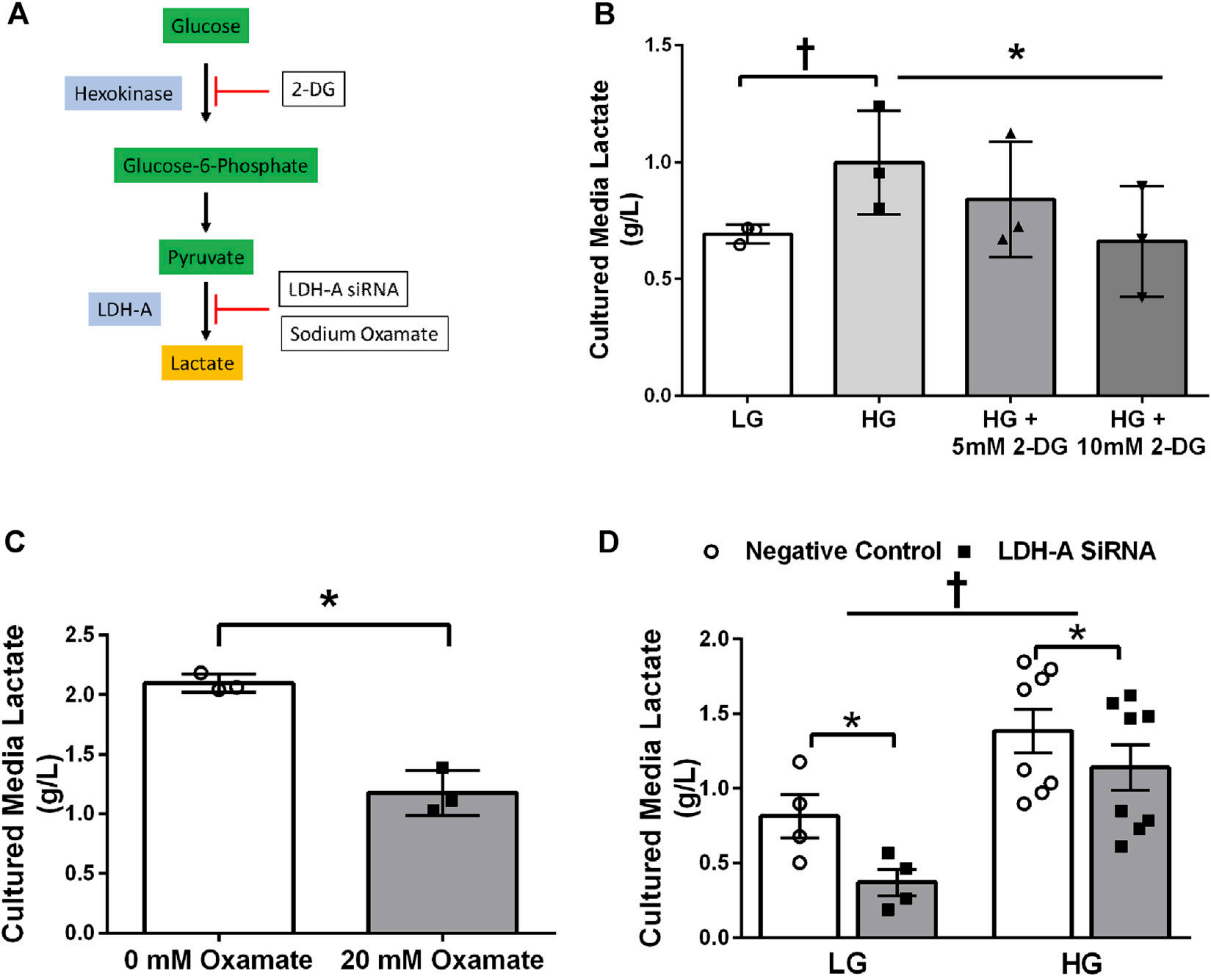
Culture Media Lactate Accumulation in Response to Glycolytic Intervention. Model of pharmacological and siRNA methodology to manipulate lactate accumulation **(A)**. Culture media lactate concentrations in response to 24 h low glucose (LG, 5.6 mmol/L) and high glucose (HG, 20 mmol/L) treatment and in response to 24 h 2-DG treatment (n = 3) **(B)**. Culture media lactate concentrations in response to 24 h sodium oxamate treatment (n = 3) **(C)**. Culture media lactate concentrations in response to negative control (Silencer Select) and LDH siRNA under low glucose (LG, 5.6 mmol/L) and high glucose (HG, 20 mmol/L) treatment (n = 8) **(D)**. Lactate concentrations were presented relative to myotube protein concentrations per well. Data is expressed as mean ± SEM. *p < 0.05 main effect for inhibitory treatment; †*p* < 0.05 low glucose vs high glucose. Sample size (n) represents biological replicates from HSkMC derived from different individuals.

**FIGURE 6:**
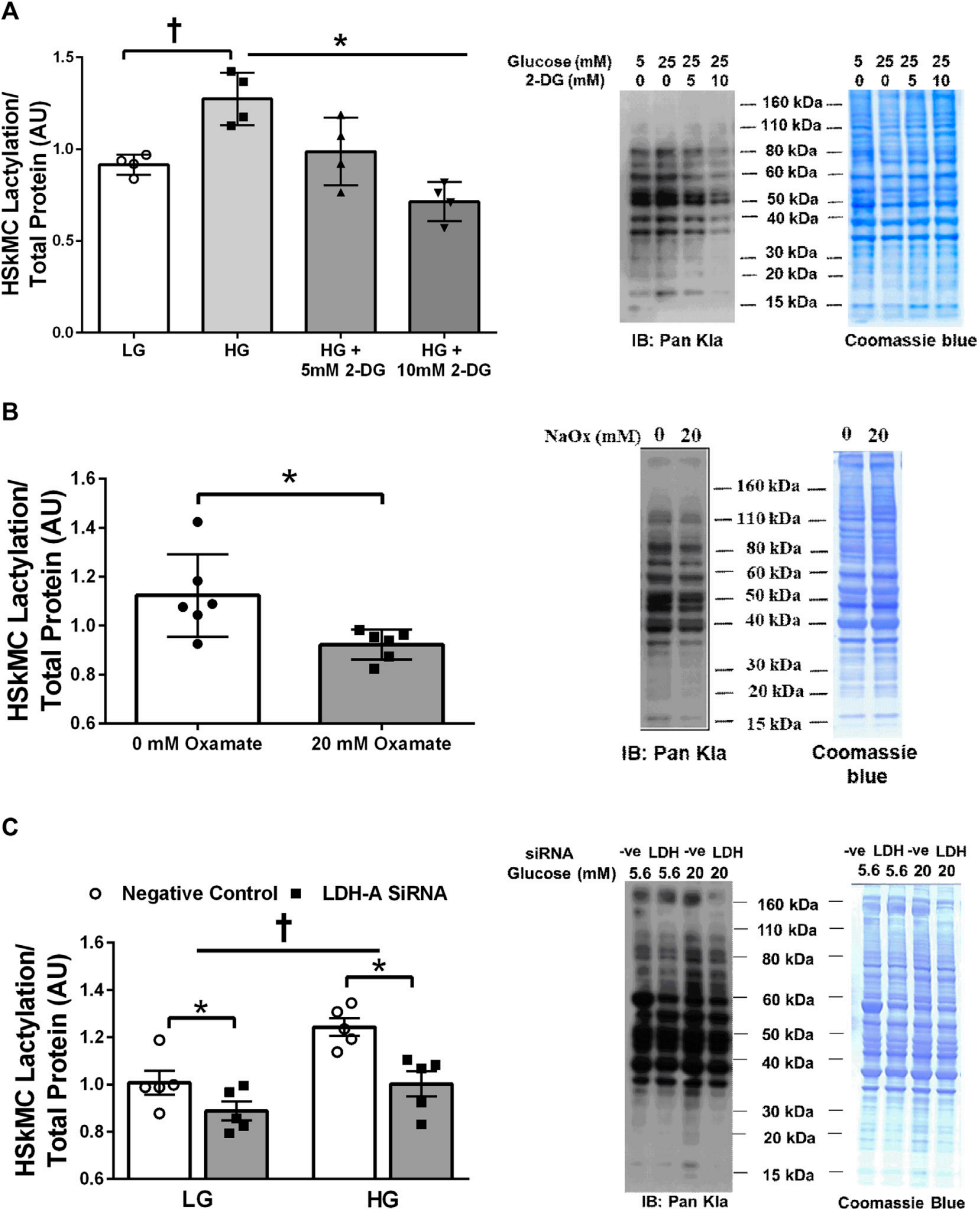
Human Primary Skeletal Muscle Cell Lactylation in Response to Glycolytic Intervention. Quantification of primary human skeletal muscle cell (HSkMC) lactylation in response to 24 h low glucose (LG, 5.6 mmol/L) and high glucose (HG, 20 mmol/L) treatment and in response to 24 h 2-DG treatment (n = 3) and representative blot for pan-kla **(A)**. Quantification of HSkMC lactylation in response to 24 h sodium oxamate treatment (n = 3) and representative blot for pan-kla **(B)**. Quantification of HSkMC lactylation in response to negative control (Silencer Select) and LDH siRNA under low glucose (LG, 5.6 mmol/L) and high glucose (HG, 20 mmol/L) treatment (n = 8) and representative blot for pan-kla **(C)**. Myotube lactylation levels were normalized to Coomassie Blue. Data is expressed as mean ± SEM. *p < 0.05 main effect for inhibitory treatment; †*p* < 0.05 low glucose vs high glucose. Sample size (n) represents biological replicates from HSkMC derived from different individuals.

Treatment of primary HSkMC with lactate for 24 h resulted in an increase in protein lactylation in a dose-dependent manner (*p* < 0.005, Figure 4). To determine the effects of endogenous lactate production on skeletal muscle lactylation, HSkMC were exposed to increased concentrations of glucose, chemical inhibitors of glycolysis and LDH siRNA technology (Figure 5A). Myotubes exposed to increased (20 vs. 5.6 mM) glucose concentrations for 24 h resulted in increased lactate accumulation (*p* < 0.05, Figures 5B,D) and increased HSkMC lactylation ∼20% (*p* < 0.05, Figures 6A,C). Twenty-four-hour treatment of HSkMC with 2-DG, a glucose analogue that acts to competitively inhibit the production of glucose-6-phosphate, reduced lactate in the culture supernatant (*p* < 0.05, Figure 5B) and reduced skeletal muscle lactylation in a dose-dependent manner (*p* < 0.01, Figure 6A). Treatment of HSkMC with the sodium oxamate, an LDH-A chemical inhibitor, for 24 h decreased both the concentrations of lactate in culture (-44%, *p* < 0.05, Figure 5C) and HSkMC lactylation (-18%, *p* < 0.05, Figure 6B). Additionally, treatment of HSkMC with LDH siRNA (decreased LDH-A protein content ∼ -55%, *p* < 0.05), decreased media culture lactate concentrations (∼-36%, *p* < 0.05, Figure 5D) and HSkMC lactylation (∼-15%, *p* < 0.05, Figure 6C) under both low (5.6 mM) and high glucose (20 mM) culture media concentrations.

## Discussion

In the current study we demonstrate for the first time that the post-translational modification, lactylation, is present in human skeletal muscle. Lactylation levels were associated with both circulating lactate and insulin resistance in humans, and tended to be higher with obesity, especially in females (Figure 1). To our knowledge, this is the first study to demonstrate lactate-induced lactylation in human skeletal muscle and provides additional insight into the relationship between lactate and insulin resistance. Previous research has reported that fasting lactate levels were associated with reduced glucose disposal rates during a hyperinsulinemic-euglycemic clamp in obese individuals (Chondronikola et al., 2018) and type 2 diabetics (Crawford et al., 2010). Furthermore, interventional studies that improve insulin sensitivity, including exercise training (Chondronikola et al., 2018; Jones et al., 2019) and bariatric surgery (Jones et al., 2019) have been found to effectively reduce blood lactate levels. Despite this knowledge, it has remained unclear if lactate accumulation was simply a byproduct of metabolic dysfunction or could act as a signaling molecule to regulate protein changes in skeletal muscle.

The present study provides new evidence for a role of lactate in regulating lactylation in human skeletal muscle. Utilizing clinical samples from lean and obese individuals, we observed a strong, positive relationship between fasting plasma lactate levels and skeletal muscle lactylation (Figure 1C). Chu et al. (Chu et al., 2021) previously reported that circulating lactate was associated with histone lactylation in the peripheral blood mononuclear cell (PBMC) of healthy and septic individuals (Chu et al., 2021). Despite *in vivo* results providing critical physiological relevance, they unfortunately do not prove insight into causation. To address whether lactate has a direct role in lactylation, we cultured primary HSkMC from human donors and observed a dose-dependent increase in myotube lactylation in response to exogenous lactate (Figure 4), similar to that reported in other cell types (Zhang et al., 2019). While our current findings do not provide a causal role of lactylation on skeletal muscle insulin resistance, it is significant to note that we also observed a lactate-induced increase in IRS-1 serine 636 phosphorylation (Figure 3A) which paralleled lactylation levels. Hyperphosphorylation of IRS-1 serine 636 has been previously reported in insulin resistant conditions (Bouzakri et al., 2003; Vlavcheski and Tsiani 2018; Den et al., 2020; Vlavcheski et al., 2020), including the HSkMC derived from type 2 diabetic donors (Bouzakri et al., 2003). Given the vital role that skeletal muscle has in regulating insulin sensitivity and the fact that previously identified post-translational modifications including phosphorylation, acetylation and malonylation have been associated with metabolic dysfunction (Tanti and Jager 2009; Du et al., 2015; He et al., 2020), the current study establishes the framework for future research to investigate the direct effects of lactylation on skeletal muscle metabolism, including insulin sensitivity.

Lactate accumulation is the net balance between lactate production and clearance. Like *in vivo* models, human skeletal muscle cells exposed to hyperglycemic conditions increase glycolytic flux and lactate production (Lund et al., 2019). Therefore, we initially investigated the effects of excess glucose availability on myotube lactylation. As expected, myotube lactylation levels were increased in response to elevations in culture glucose (Figures 6A,C). In contrast, addition of 2-DG, an upstream inhibitor of glycolysis, diminished endogenous lactate accumulation (Figure 5B) and reduced myotube lactylation (Figure 6A). A minor limitation of the current study was that we did not investigate the impact of lactate transport on lactylation levels and that the measurement of culture lactate was likely the product of changes in both lactate production and lactate oxidation rates, especially during hyperglycemic conditions (Lund et al., 2019). Regardless, the focus of the current study was to investigate lactylation levels in response to conditions that promoted lactate accumulation. Taken together, these results demonstrate that skeletal muscle lactylation levels increase in response to elevated glycolytic flux and lactate accumulation, similar to that reported in other cell types (Zhang et al., 2019; Yu et al., 2021).

There is clear evidence that the intracellular fate of glucose becomes dysregulated during insulin resistance. The preferential shuttling of glucose to lactate at the expense of oxidation or glycogen storage have been well documented in insulin resistant conditions (Shulman et al., 1990; Consitt et al., 2016; Zou et al., 2019). It is thought that the imbalance of pivotal intracellular enzymes or pathways may contribute to this favored shuttling of glucose towards lactate. For example, impaired mitochondrial TCA flux (Zou, Hinkley et al., 2019) and reduced PDH function (Consitt et al., 2016) have been suggested as potential contributors to the enhanced pyruvate to lactate conversion in insulin resistant conditions. Given this knowledge, it is not surprising that we found that individuals with lower skeletal muscle COXIV expression (mitochondrial protein) had higher levels of skeletal muscle lactylation (Figure 2B). Additionally, we observed a strong negative relationship between skeletal muscle lactylation levels and the rate of complex I (pyruvate supported) OXPHOS capacity in permeabilized muscle fibers from a subset of subjects (Figure 2D). In contrast, we found that skeletal muscle lactylation was positively associated with skeletal muscle expression of LDH-A (Figure 2A), the protein responsible for the conversion of pyruvate to lactate. The role of LDH-A in lactylation was further strengthened when we demonstrated that decreasing LDH-A expression (via siRNA technology) or inhibiting LDH-A activity (via sodium oxamate) in human myotubes was sufficient to decrease protein lactylation (Figures 6C and B, respectively). Together, these results support the notion that individuals with an enhanced skeletal muscle anaerobic to oxidative capacity ratio would be at risk for excess lactate accumulation and potentially enhanced skeletal muscle lactylation.

The goal of the current study was to determine if lactylation was present in human skeletal muscle utilizing a pan anti-Kla antibody commonly used to identify lactylation in other cell types (Zhang et al., 2019; Chu et al., 2021; Cui et al., 2021; Hagiharai et al., 2021; Jiang et al., 2021; Meng et al., 2021). The confirmation of lactate-induced lactylation in human skeletal muscle advocates for future research to identify the specific proteins that undergo lactylation. Lactate-induced lactylation was first discovered on the histones of M1 macrophages (Zhang et al., 2019) with subsequent studies continuing to focus on these nuclear proteins (Chu et al., 2021; Cui et al., 2021; Jiang et al., 2021; Pan et al., 2022). Histones consist of a central histone fold and lysine-rich tails that undergo various post-translational modifications that affect chromatin structure and transcription (Li and Delaney 2019). While the current study did not directly measure histone lactylation, it is reasonable to assume that the lactate-induced lactylation of the 17 kDa molecular weight protein (Figure 4) was a histone protein. To help validate this assumption, we stripped membranes and reprobed with a Histone H3 antibody and produced a band of similar molecular weight (17 kDa). Conditions that stimulate glucose uptake including exercise and insulin stimulation have been proven to affect other post-translational modifications of histones in skeletal muscle (Kabra et al., 2009; McGee et al., 2009; Pandorf et al., 2009; Zheng et al., 2012; Lim et al., 2020) and skeletal muscle histone acetylation and methylation have been reported in diabetic animal models (Yonamine, et al., 2019), highlighting the susceptibility of histones to these modifications in skeletal muscle.

More recently, lactylation of non-histone proteins been reported in both the plant fungus *Botrytis cinerea* (Gao et al., 2020) and developing rice (Meng et al., 2021). Global profiling of the lysine lactylome in developing rice identified a total of 638 lactylation sites on 342 proteins and that approximately 33% and 10% of these lactylated protein were located in the cytoplasm and mitochondria, respectively. Furthermore, a large portion of enzymes located within the glycolytic and TCA cycle pathway contained lactylated sites, including PDH, citrate synthase, and malate dehydrogenase (Meng et al., 2021). While it remains unknown what role, if any, lactylation may have on the function of these proteins, it is intriguing that several proteins associated with insulin resistance also contain lactylation sites, at least in rice. These global lactylome findings combined with our present findings warrant future research to identify the lactylated proteins in skeletal muscle and determine the physiological significance of this post-translational modification.

In summary, the findings from the current study demonstrate for the first time that lactylation is present in human skeletal muscle and is associated with circulating lactate and insulin resistance in humans. Supporting our *in vivo* findings, we observed lactate-induced lactylation in primary HSkMC which paralleled increases in IRS-1 serine phosphorylation. Furthermore, inhibiting the glycolytic pathway, which is often upregulated in insulin resistant conditions, decreased human myotube lactylation levels. The current study provides the framework for future studies to identify the specific proteins that undergo lactylation in skeletal muscle and determine the metabolic consequences of this new post-translational modification in skeletal muscle.

## Data Availability

The original contributions presented in the study are included in the article/supplementary material, further inquiries can be directed to the corresponding author.
